# Testing the art as adaptation hypothesis through artistic practice and reproductive success in Papua woodcarvers

**DOI:** 10.1038/s41598-025-06394-y

**Published:** 2025-07-01

**Authors:** Piotr Sorokowski, Marta Kowal, Jerzy Luty, Wiktoria Jędryczka, S. Craig Roberts, Anjan Chatterjee, Stephen Davies

**Affiliations:** 1 Department of Psychology, Institute of Psychology, University of Wroclaw, ul. Dawida 1, 50-527 Wroclaw, Poland; 2 Being Human Lab, University of Wroclaw, 50-527 Wroclaw, Poland; 3 Department of Psychology, University of Stirling, Stirling, FK9 4LA UK; 4 Department of Neurology, Perelman School of Medicine, University of Pennsylvania, Philadelphia, PA 19104 USA; 5 Faculty of Arts, University of Auckland, Auckland, 1010 New Zealand

**Keywords:** Artists, Reproductive success, Creativity, Conscientiousness, Aesthetics, Asmat, Papua, Human behaviour, Social anthropology

## Abstract

Archaeological studies provide evidence that art was a well-established activity among *Homo sapiens* as early as 50,000 to 75,000 years ago. Today, people continue to spontaneously create artistic works from an early age, and some master artistic skills with such proficiency that they become known as artists. Considering the ubiquitous nature of art, many evolutionary-oriented scholars have pondered the potential role of art, proposing the ‘art as an adaptation hypothesis’, according to which art serves social functions that promote the survival and reproductive success of individuals. Surprisingly, very few attempts have been made to verify this hypothesis empirically. Here, we addressed this issue, investigating whether art can be an adaptation: that is, whether it can increase the reproductive success of the artist. We collected data from indigenous inhabitants of Papua (Asmat and Kamoro societies), including 101 self-described carvers (artists) and 130 non-artists. Unadjusted models suggested that Asmat and Kamoro artists have higher reproductive success (measured by the number of their children), although this association disappeared in models adjusted for potentially relevant confounds. We also found that the relationship between reproductive success and being an artist can be partly explained by artists’ higher conscientiousness and creativity. While it would be plausible to explain artistic behaviour as an adaptation, the data are also compatible with such behaviours being by-products of an adaptation.

**Supplementary Information:**

The online version contains supplementary material available at 10.1038/s41598-025-06394-y.

## Introduction

The human interest in art seems to have deep historical and biological roots. Archaeological discoveries reveal early manifestations of artistry and skilled craftsmanship, such as engraved shells and precisely shaped hand-axes created by *Homo erectus* approximately 400,000–500,000 years ago^1,2^. Neanderthals also contributed to the artistic legacy, leaving evidence of their creativity in forms like cave paintings that date back 64,000 years^3^. With early *Homo sapiens*, artistic expression flourished further; some of the oldest engravings and drawings, found in South Africa, illuminate this expanding creative trajectory^4–6^. Remarkably, the oldest figurative image and a detailed hunting scene – dating back more than 40,000 years – were both unearthed in Indonesia, underscoring the ancient artistic traditions within this region, which includes the Pacific islands examined in this study^7,8^.

Traces of art are widespread throughout the centuries and are easily recognised in all historical eras^9,10^. They are also geographically widespread, occurring along the Arctic coasts inhabited by Inuits, and in the drylands of Africa, humid Asian territories, and isolated Pacific islands^2,10^. Furthermore, the fact that very young children (e.g. 2-year-olds) create songs, paint, and draw indicates that the desire to create art might even be innate^11–13^. Based on all the evidence above, one can at least conclude that art may have served a role in the evolution of the human species^14–17^.

Darwin^18^ noticed that many male animals (including humans) possess various psychological traits and physical ornaments that, he suggested, evolved as adaptations due to female mate preferences. Following this logic, many scholars have attempted to explain the ubiquitous nature of art by referring to its adaptive role (see below), as well as through other mechanisms, such as costly signalling and increasing group cohesion/cooperation. According to the costly signalling theory (as discussed by Hodgson^6^ and others), certain behaviours or traits – including art – may serve as signals of individual quality or commitment, despite their high cost. This perspective has been applied to art in both archaeological and contemporary contexts, suggesting that artistic expression may serve not only as an adaptive trait but also as a costly signal within social or mating contexts^19^. Furthermore, it may also help explain the links between art, religious behaviours, and costly signalling (e.g^20,21^). Art could also play a crucial role in fostering group bonding, cooperation, and coalition formation, as evidenced by numerous anthropological studies^22–24^. In many traditional societies, collective artistic activities strengthen social ties and promote group cohesion; this social function has been best verified in the fields of music and dance^25,26^.

Finally, one of the most critical questions in evolutionary aesthetics concerns the hypothesised adaptiveness of art, such that being an artist might increase reproductive success through the mechanism of sexual selection^16,17^. Of course, the adaptiveness of art may depend on the specific context in which it is expressed: art could potentially serve adaptive functions in some environments but not in others. Moreover, while art in its various forms may be an adaptation, it could also be considered a by-product of adaptation (i.e. the spandrel hypothesis^27^), or might not be connected to evolutionary adaptation at all. Many distinguished authors (e.g^14,16,17,28–30^), and many other philosophers, biologists, cognitivists, evolutionary psychologists, and art historians have discussed this matter at length, proposing many hypotheses. Nonetheless, there has been relatively little empirical research on art from an evolutionary perspective. Therefore, most of the hypotheses formulated in evolutionary art theory remain speculative and are not empirically driven explanations based on methodologically reliable observations. In other words, we still do not know whether the evidence supports or refutes such ideas.

The lack of research explicitly testing the adaptive role of practising art and its impact on artists’ reproductive potential may seem surprising. There is only very limited evidence that, at least in Western communities, artists may have more sexual partners or be perceived as more desirable partners for a hypothetical date (e.g^31^). On the other hand, research addressing the hypothesis that being an artist positively influences the overall assessment of attractiveness as a potential partner has yielded mixed results (see^32,33^). Similarly, a study from the field of behavioural genetics found little support for the adaptive hypothesis. Analyses using twin modelling in a large sample (< 10,000 twins) revealed no significant relationships between musical ability and measurements of mating success. However, there was a very weak association (*r* = 0.09) between musical achievements in men and their number of children^34^. Moreover, in the only study that examined a society with natural fertility, creative potential (this study did not analyse artistic abilities) negatively predicted the number of offspring^35^.

To sum up, no research (except for the studies of musical abilities in twins^34^) has directly tested whether artistic skills might affect human reproductive success, by using (for example) data from parish records or by studying natural fertility populations.Moreover, even if artistry is correlated with reproductive success, this could be explained by alternative mechanisms. We can distinguish many features that are not adaptations, such as spandrels or by-products of other adaptations^27,36–38^. Although this concept is sometimes criticised, many scientists use it to explain the results of their research and analyses^39–43^.

In the case of art, we could speculate that artistic abilities were not directly adaptive but appeared as an addition to cognitive skills that determine survival and reproduction. Many authors, including Pinker^30,44^, support or consider such a possibility, surmising that aesthetic sensitivity is merely a side effect of the cognitive abilities that evolved to fulfil more practical functions, such as the pursuit of status or the ability to produce objects of practical use.

**Fig. 1:**
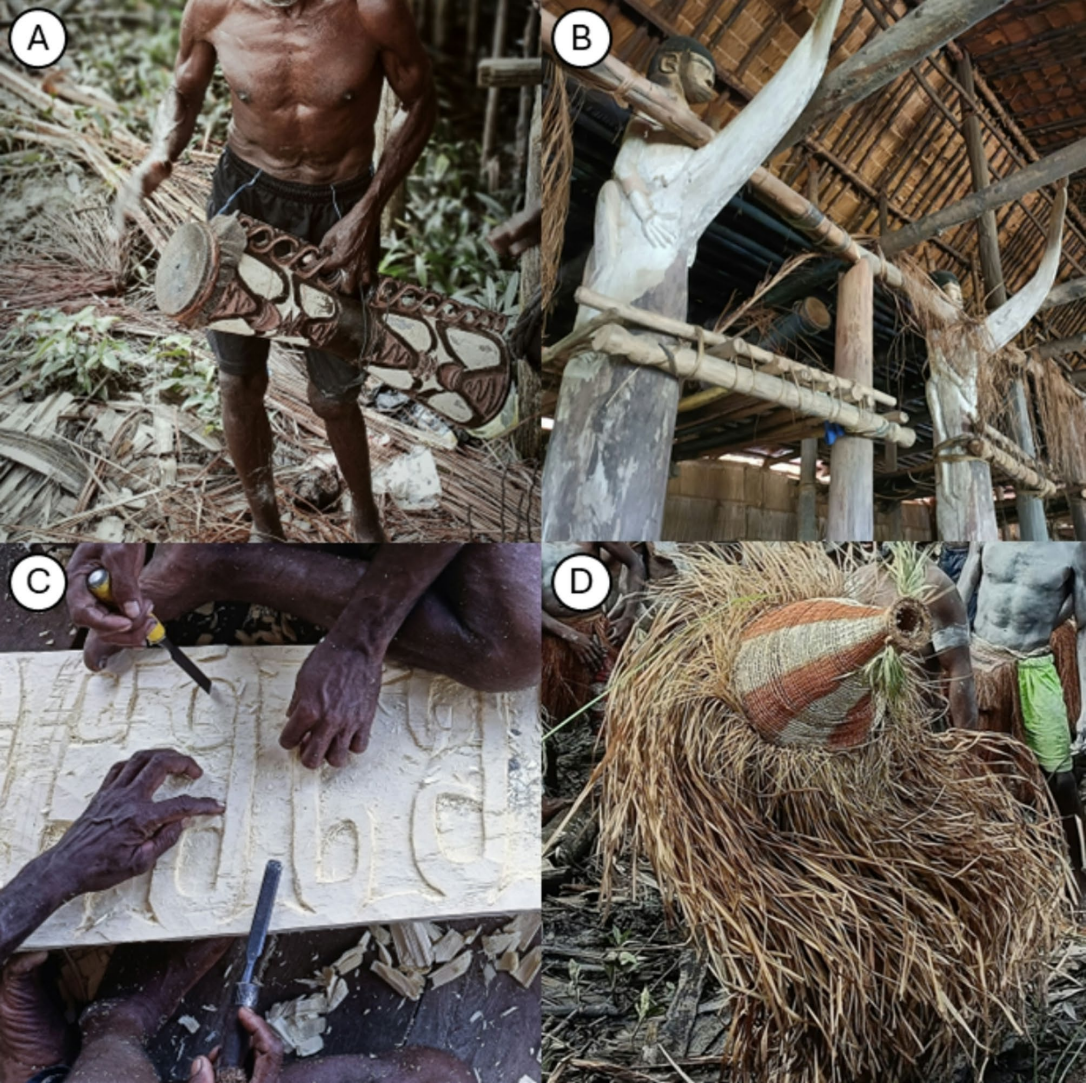
Examples of artistic products in Asmat and Kamoro societies: (**A**) Traditional drum; (**B**) Vertical posts supporting roofbeams, in which the support is in the shape of a warrior with large pointed phallus; (**C**) Shield work; (**D**) Ritual mask.

**Fig. 2:**
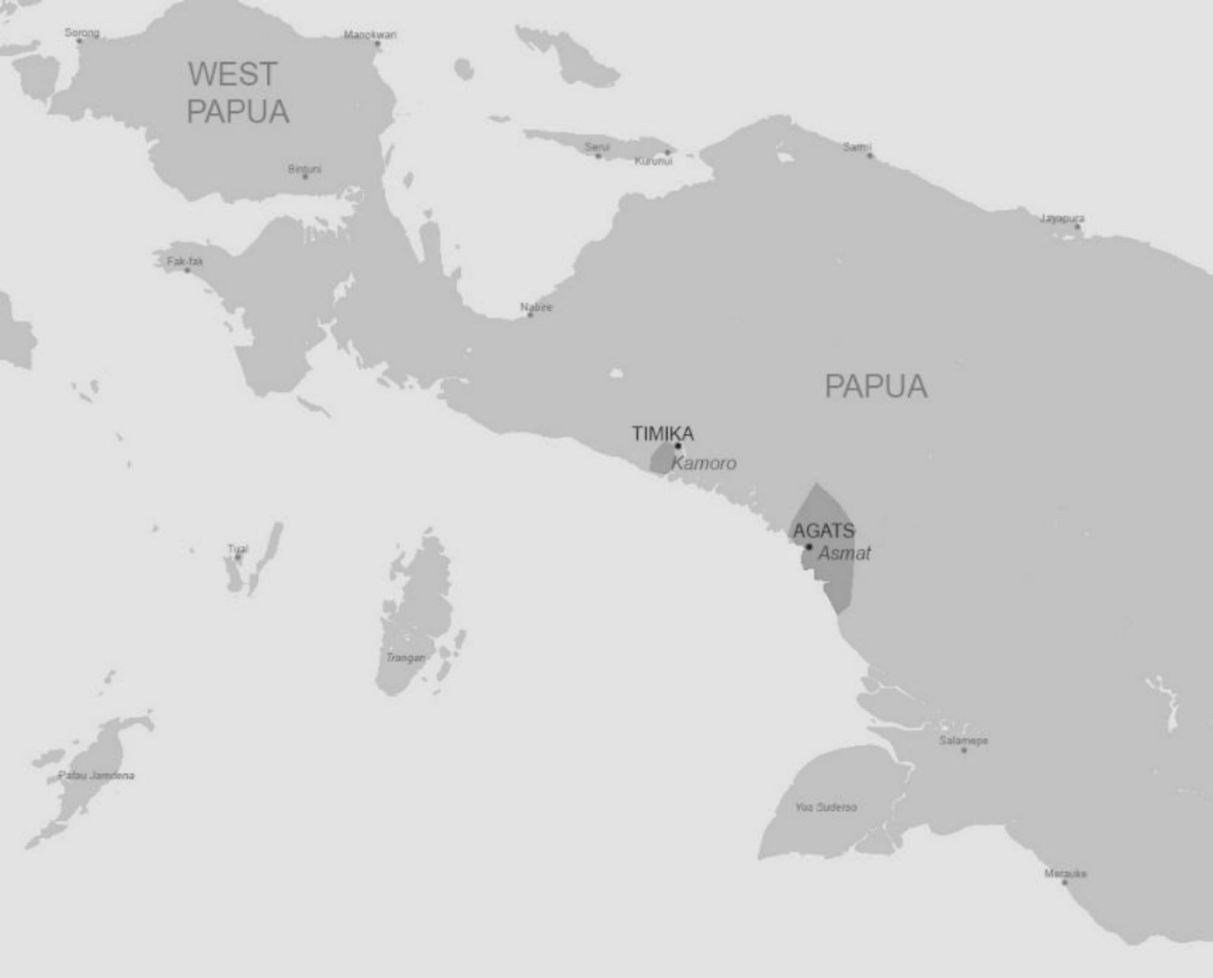
Map showing the regions (highlighted in dark grey) where the study was conducted. The map has been drawn in Paint by WJ (https://jspaint.app/#local:37bae72758def*).*

We explored these predictions by conducting an empirical study among members of two traditionally living groups – the Asmat and Kamoro people from Papua – who are well-known for their works of art (see Fig. 1), in particular carving^45,46^. The two societies are related, both using the Asmat–Kamrau Bay languages^47^ and living in very similar environmental conditions (see Fig. 2). To better understand the context of our study, it is worth mentioning some ethnographic information. Both groups traditionally practice monogamy, although serial monogamy and remarriage are common due to high mortality rates. Polygyny is rare and typically limited to high-status individuals in some subgroups. These patterns suggest that mate access is not entirely equal across individuals and may play a mediating role in reproductive success. Traditionally, the Asmat and Kamoro communities have engaged in foraging, fishing, small-scale hunting, and – where possible – horticulture (e.g. cultivating sago palm). Today, some individuals participate in wage labour, and the occasional sale of artistic products, such as woodcarvings, reflects a partial integration into the market economy. While both societies retain substantial cultural autonomy, external influences such as tourism have facilitated some connection with the market economy (this is not mass tourism but rather visits by a few people who make the effort to reach these remote areas to explore the unique culture and natural beauty of Papua). Both groups maintain hierarchical social structures in which elders and individuals with specialised skills – including master woodcarvers – have high prestige. Asmat and Kamoro woodcarvings hold significant social, ritual, and symbolic meaning. Traditionally, carvings are central to initiation rites and other communal events. While many of these works are now sold to tourists and collectors as a source of income, the primary purpose of creating art is not commercial. In Asmat and Kamoro villages, one can find an abundance of indigenous art, including everyday objects and decorations on and within homes. The most famous carved Asmat and Kamoro objects include ceremonial spirit poles (called *bjis pole* in Asmat, and *mbitoro* in Kamoro), human figures, and animal figures, as well as decorated shields, musical instruments, canoes, and tools (see Fig. 1). Some well-known museums that have collections of Asmat and Kamoro art include the Metropolitan Museum of Art in New York, the National Museum of Ethnology in Leiden, the National Museum of Natural History in Paris, and the British Museum in London. Importantly, both of these communities were producing art long before they were first encountered by Westerners^46^.

Mastering woodcarving requires years of dedicated practice, typically through an apprenticeship system, where a master not only imparts technical skills but also the symbolic knowledge embedded in the art. Carving is considered a male occupation in this community. Consequently, our study’s sample consisted solely of men. Becoming a carver is open to any man who shows the desire and basic skills; we often observed individuals who were encouraged by their community members to try learning to carve. This is noteworthy in the context of our study because, for instance, conducting similar research in a society where the position of an artist is inherited from father to son (like *griots* in West Africa) would preclude a proper test of our hypothesis. For our study, it was crucial that these communities had a substantial number of artists (carvers), which made it easier to assemble a large sample group. Woodcarvers are highly respected within their communities, as their work reflects both technical skill and a deep connection to cultural beliefs and identity. In addition to woodcarving, Asmat and Kamoro art includes body painting and ornament production (such as ritual masks), each serving various purposes, from decorative to ritualistic. Although the two groups share many cultural similarities and a closely related language, they differ in artistic styles and certain ceremonial practices. However, they are united by the prominence of artists within their societies, the high prestige of woodcarvers, and the abundance of traditional artworks found throughout their villages.

Along with testing the direct link between art and reproductive success, we also investigated the additional explanation, that is, whether this hypothesised link could be explained by other traits that are potentially more directly linked to reproductive outcomes. In such a case, it would be more parsimonious to conclude that art is a by-product of these other adaptive characteristics. Based in part on the literature review and our observations and discussions with the Papuan people, we hypothesised that such other adaptive characteristics might be conscientiousness, creativity, and visuo-motor coordination, which are often associated both with high artistic skills^48,49^ and higher survival and reproduction. In other words, we observed that, for example, conscientiousness is important for local carvers, because people who do not want to work and who take breaks from carving (presumably because they lack conscientiousness) are poor sculptors or are not sculptors at all. At the same time, many successful people, not necessarily artists, tend to have high levels of conscientiousness^50^. Similarly, creativity is an obvious example of a trait that is useful not only for artists but also for every human being^51^, and visuo-motor skills may be helpful in activities such as fighting or hunting, as well as in creating art^52^.

## Methods

### Participants

To determine a sample size with power 0.95 and 0.05 alpha error probability to detect medium effects in a mediated effects analysis, we used a Monte Carlo power analysis simulation (1,000 replications) and tested the indirect effect with bootstrapped confidence intervals. Results revealed that a sample size of *n* = 230 should be sufficient to detect a medium (*r* = 0.30) size indirect effect with power 0.95 and 95% CI[0.93, 0.97]. This number is also consistent with the usually recommended criteria for mediation analyses^53^, which (using the conservative rule) advise using a sample size of ~ 224 participants for detecting medium effects. Furthermore, such a sample size would also be sufficient to detect a small to medium (f_2_ = 0.1) effect size in a linear regression with power 0.99.

We recruited 231 male members of the Asmat (*n* = 131) and Kamoro (*n* = 100) peoples, indigenous inhabitants of West Papua (an Indonesian province). The research was conducted during three field studies in 2019–2023 by the first author and a local assistant. The study was conducted in the vicinity of the villages Kamora, Espe, Uus, Per, Amborep, and Warse. We interviewed only men because only men are carvers in these communities. Every man who wished to participate was able to join the study. As is typical in studies of this nature, we announced the opportunity and encouraged people to take part, with individuals signing up voluntarily. Due to the targeted sampling, we included every artist who expressed interest; in practice, the sample of non-artists could have been larger. We estimate the rejection rate for invitations in this type of study to be around 20–25%. The subjects were invited to participate in a study in which we discussed local art and their lives. We informed them that they could withdraw from the study at any time. During the meetings, we also asked other questions unrelated to the project, conversing about Papuan culture, art, and their lives. Our sample consisted of 101 self-described carvers and 130 non-carvers [later in the article described as artists and non-artists]. Their age ranged from 18 to 70 years old (*M* = 36.78, *SD* = 11.95; *M*_*artists*_ = 38.01, *SD*_*artists*_ = 11.21; *M*_*non−artists*_ = 35.82, *SD*_*non−artists*_ = 12.45). Participants’ number of children ranged from 0 to 10 (M = 3.99, *SD* = 2.31; *M*_*artists*_ = 4.45, *SD*_*artists*_ = 2.37; *M*_*non−artists*_ = 3.64, *SD*_*non−artists*_ = 2.20).

Participants were compensated for their participation (equivalent to approximately US$5). The study was conducted following the recommendations of the Declaration of Helsinki. The Institutional Ethics Committee at the Institute of Psychology of the University of Wroclaw approved the study’s protocols and gave ethical approval for conducting the study. The study was also approved by the local community leaders. The local assistant was involved in data collection and was employed in the project. Local people participated in the project by contributing to the development of the methodology and engaging in discussions to help identify which variables should be included in the study (mediation model). Original data are available and can be accessed: https://osf.io/tfxjd/?view_only=590af1935a124c6dbf7babba00fd77ee.

### Materials and measures

We measured participants’ reproductive success by the number of their children (“children born to the participants”, according to information received from the participants). Conscientiousness was measured using the conscientiousness subscale of the classical psychological personality test, the NEO-FFI^54^. The response scale ranged from 1–*strongly disagree* to 5–*strongly agree*. The scale was highly reliable (Cronbach’s *α* = 0.88, McDonald’s *ω* = 0.90). To facilitate the task for our subjects, who probably had never completed a single questionnaire in their lives, we did not use flipped questions. In our study, the original flipped questions from the scale^54^ were: “I don’t waste any time, I go to work right away”, “I’m reliable as I should be”, and “I’m always well organised”. The question “I am not a very methodical person” was difficult to translate and incomprehensible to the respondents, so it was not used. Creativity was assessed with the Alternative Uses Task (AUT;^55^), a widely recognised and popular tool for assessing creativity. It requires participants to generate multiple unique uses for a given object (in the present case, a stone) within a given timeframe (30 s); the number of uses was summed to calculate a participant’s creativity score. Visuo-motor coordination was measured with the Purdue Pegboard Test (PPT;^56^), a psychomotor test to assess manual dexterity and bimanual coordination. This test measures two distinct abilities, namely, gross movements of the arms, hands, and fingers and fine motor control, also known as “fingerprint” dexterity. A subpar performance on the Pegboard test indicates difficulties with visually guided and coordinated movements that require more intricate and complex movements^57^. Right- and left-hand scores were averaged to create a visuo-motor coordination score.

### Procedure

The participants were tested individually in their natural settings and informed, prior to participation, about the general goals of the study. All participants provided informed consent in oral form. The decision to obtain verbal rather than written consent was guided by the nature of the research and the cultural context of the participant population. The study was conducted entirely through interviews, making verbal communication the primary mode of interaction. Moreover, a significant portion of the participants had limited or no literacy skills, which made obtaining written consent impractical and potentially intimidating for them. The study was conducted in the Indonesian language, which our participants understood fully. In a brief pre-test involving approximately ten participants, we ensured that the questions in our questionnaire were entirely understandable to the respondents.

### Data analyses

In the first step, artists and non-artists were compared in terms of age distributions with a *t*-test. Next, the normality of the outcome variable (i.e. reproductive success measured by the number of participants’ children) was investigated by adhering to standard guidelines regarding skewness and kurtosis^58^. Then, two linear regression models were assessed. The first included reproductive success (i.e. the number of children a participant had) as an outcome variable and a dichotomous variable of being an artist or not (with non-artists coded as 0 and artists as 1) as the predictor variable. The second model included the additional variables, that is, conscientiousness (mean score on the conscientiousness scale), creativity (the total amount of generated uses for a stone), and visuo-motor coordination (mean of the right- and left-hand score on the visuo-motor coordination test). Participants’ age (centred) was controlled for in both models. The two models were then re-run, this time controlling for a curvilinear relationship between the number of children and age (centred); this adjustment allows for detecting cohort-specific influences on the number of children that lie outside the primary hypothesised links (for example, historical events like famine or war that might have reduced reproductive success in men of certain ages compared with older or younger cohorts).

A multiple mediation analysis with a maximum likelihood estimator was performed in the following step. As before, the predictor variable was being an artist, the outcome variable was reproductive success, and the mediators were conscientiousness, creativity, and visuo-motor coordination. In this model, correlations between mediators were allowed. Again, we first controlled for the participants’ age, and in the subsequent model, we controlled for participants’ age and age squared. For ease of interpreting the results and comparing the effect sizes, we applied the post-hoc standardisation.

In summary, the linear models that include age, conscientiousness, creativity, and coordination as covariates assume that these variables are confounders of the relationship between being an artist and reproductive success. Therefore, they need to be adjusted for when assessing this relationship to reduce bias due to potential confounding. In contrast, the models that treat conscientiousness, creativity, and coordination as mediators assume that these variables represent mechanisms through which artists may achieve greater reproductive success (e.g. being an artist increases creativity, which in turn influences reproductive success). Given the cross-sectional nature of our data, we cannot distinguish between these two alternatives. From a theoretical perspective, both seem plausible, so we present both models.

All the analyses were performed in R software (version 4.2.0), using the following packages: *dplyr*^59^, *e1071*^60^, *lavvaan*^61^, *parameters*^62^, and *psych*^63^.

## Results

The *t*-test did not provide evidence for any age differences between artists and non-artists (*t*_(231)_ = − 1.401, *p* = 0.163, Cohen’s *d* = − 0.180). The outcome variable was within the expected ranges of the skewness values below |2| and kurtosis values below |7^58^. The distributions of the number of children across the entire sample and separately for artists and non-artists are presented in Figs. 1, 2 and 3 in the Supplementary Materials. The first regression model (Table 1) showed a significant link between the number of children (a proxy of reproductive success) and being an artist (*β* = 0.134, *SE* = 0.059, *p* = 0.023). The results of the unadjusted model suggest that being an artist is positively related to increased reproductive success.

This relationship was not significant in the second model (β = − 0.034, SE = 0.063, *p* = 0.590), when the variances of conscientiousness, creativity, and visuo-motor coordination were accounted for (see Table 1). This result suggests that conscientiousness, creativity, and visuo-motor coordination may confound the relationship between reproductive success and being an artist. In subsequent models, when we introduced age squared (testing the potentially curvilinear relationship between age and the number of children) and society (i.e. Asmat and Kamoro), being an artist was not significantly related to the number of children (see Table S1 in the Supplementary Materials).

**Fig. 3:**
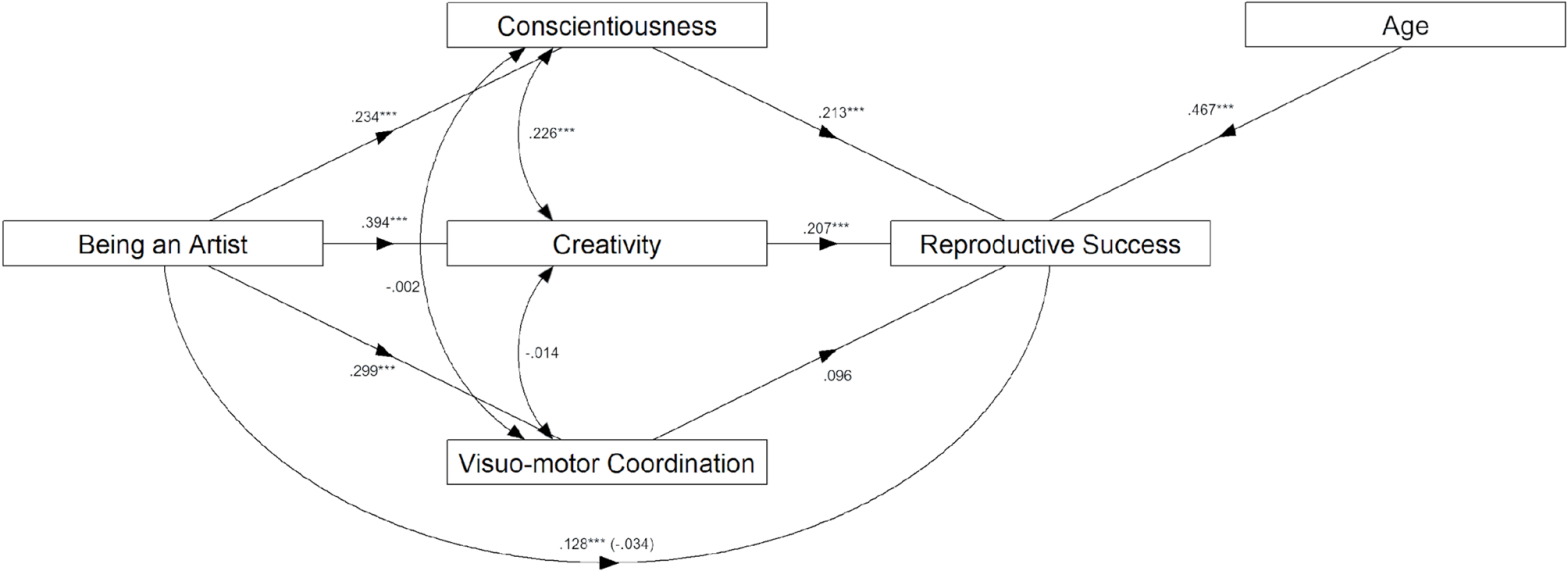
A visual representation of the multiple mediation model (standardised coefficients) with the outcome variable being reproductive success (i.e. the number of children), the predictor variable being an artist (non-artists coded as 0), and three mediators (conscientiousness, creativity, and visuo-motor coordination), controlling for participants’ age. *Note* * *p* < 0.05, ** *p* < 0.01, *** *p* < 0.001.

In the mediation models, we assessed a second possibility: that the three variables (i.e. conscientiousness, creativity, and visuo-motor coordination) are not confounders but mediators of the link between being an artist and reproductive success. The results of the mediation model showed that the association between being an artist and reproductive success was no longer significant after introducing the proposed mediators, suggesting a possible case of full mediation. More specifically, being an artist was positively associated with both conscientiousness and creativity, which, in turn, were positively associated with reproductive success. These findings suggest that while being an artist is initially associated with having more children, that association appears to operate through higher conscientiousness and creativity among artists. However, the mediation model controlling for the curvilinear relationship between age and the number of children did not yield the same results. Specifically, there was no evidence for a link between being an artist and the number of children when controlling for age and age squared (see Table 2). A visual representation of the multiple mediation model is presented in Fig. 3.

**Table 1:** The results of the linear regression models, with reproductive success (i.e. the number of children) as the outcome variable and being an artist as a predictor variable in the first model, and being an artist, conscientiousness, creativity, and visuo-motor coordination as predictor variables in the second model, controlling for age and age squared.

Predictor		Adj. *r*^2^ = 0.222, *F*(2,228) = 31.9, *p* < 0.001***						Adj. *r*^2^ = 0.310, *F*(5,225) = 21.7, *p* < 0.001***				
		b		β	SE	95% CI	*p*	B	β	SE	95% CI	*p*
Models controlling for a linear relationship between the number of children and age												
Being an artist ^a^		0.623		0.134	0.059	[0.018, 0.250]	0.023*	−0.159	−0.034	0.063	[−0.159, 0.091]	0.590
Age		0.084		0.436	0.059	[0.320,0.552]	< 0.001***	0.092	0.475	0.056	[ 0.365, 0.585]	< 0.001***
Conscientiousness ^b^								0.675	0.217	0.058	[ 0.103, 0.331]	< 0.001***
Creativity ^c^								0.685	0.211	0.061	[ 0.089, 0.332]	< 0.001***
Visuo-motor Coordination ^d^								0.108	0.098	0.058	[−0.016, 0.212]	0.093

Predictor		Adj. r^2^ = 0.233, F(3,227) = 24.26, *p* < 0.001***						Adj. r^2^ = 0.333, F(5,225) = 20.16, *p* < 0.001***				
		b	β		SE	95% CI	*p*	B	β	SE	95% CI	*p*
Models controlling for a curvilinear relationship between the number of children and age												
Being an artist ^a^		0.518	0.111		0.059	[−0.004, 0.227]	0.058	−0.268	−0.058	0.063	[−0.182, 0.066]	0.359
Age		0.099	0.511		0.064	[ 0.384, 0.638]	< 0.001***	0.107	0.553	0.061	[ 0.433, 0.673]	< 0.001***
Age^2^		−0.003	−0.173		0.064	[−0.300, − 0.046]	0.008**	−0.003	−0.179	0.061	[−0.298, − 0.059]	0.004**
Conscientiousness ^b^								0.711	0.229	0.057	[ 0.116, 0.341]	< 0.001***
Creativity ^c^								0.632	0.194	0.061	[ 0.075, 0.314]	0.002**
Visuo-motor Coordination ^d^								0.121	0.110	0.057	[−0.002, 0.222]	0.055

*p < 0.05, ** *p* < 0.01, *** *p* < 0.001; ^a^ a dichotomous variable of being an artist or not (with non-artists coded as 0 and artists as 1); ^b^ a mean score on the conscientiousness scale; ^c^ the total amount of generated uses for a stone; ^d^ a mean of the right- and left-hand scores on the visuo-motor coordination test.

**Table 2:** A summary of the multiple mediation analysis.

Relationship	Direct effect				Indirect effects			
	Unstandardized estimate	SE	95%CI	*p*	Unstandardized estimate	SE	95%CI	*p*
Model (controlling for a linear relationship between age and the number of children)								
Artists ^a^ → Conscientiousness ^b^ → Reproductive Success ^c^	0.606	0.271	[0.075,1.137]	0.025*	0.245	0.091	[0.067,0.423]	0.007**
Artists ^a^ → Creativity ^d^ → Reproductive Success ^c^					0.385	0.125	[0.140,0.630]	0.002**
Artists ^a^ → Visuo-motor Coordination ^e^ → Reproductive Success ^c^					0.135	0.084	[−0.030,0.300]	0.105
Model controlling for a curvilinear relationship between age and the number of children								
Artists ^a^ → Conscientiousness ^b^ → Reproductive Success ^c^	0.498	0.270	[− 0.031,1.027]	0.065	0.258	0.093	[0.076,0.440]	0.005**
Artists ^a^ → Creativity ^d^ → Reproductive Success ^c^					0.355	0.121	[0.118,0.592]	0.003**
Artists ^a^ → Visuo-motor Coordination ^e^ → Reproductive Success ^c^					0.152	0.083	[−0.011,0.315]	0.068

* *p* < 0.05, ** *p* < 0.01, *** *p* < 0.001. ^a^ a dichotomous variable of being an artist or not (with non-artists coded as 0 and artists as 1); ^b^ a mean score on the conscientiousness scale; ^c^ the number of children; ^d^ the total amount of generated uses for a stone; ^e^ a mean of the right- and left-hand scores on the visuo-motor coordination test, *r*^2^ for the model with a linear relationship with age = 0.349, *r*^2^ for the model with a curvilinear relationship with age = 0.375.

## Discussion

For many years, scientists have discussed the prevailing human tendency to create and admire art^16,17,30,64,65^. One potential explanation for this phenomenon involves the adaptive role of art. An adaptation refers to a hereditary trait resulting from natural selection that aided the survival or reproductive success of ancestors. It has been proposed that artistic prowess, similar to the peacock’s tail^66^, could serve as a costly signal for sexual display, attracting potential mates. Individuals capable of mastering art skills and dedicating significant time to artistic pursuits might thus signal their high genetic quality^28^.

Our analyses yielded mixed results, indicating that they should be interpreted with caution. In the regression models, we examined the association between being an artist and reproductive success, while testing whether other variables might act as confounders in this relationship. In a basic model, we found a significant positive link between being an artist and the number of children, suggesting that artists might achieve higher reproductive success. However, this relationship was no longer statistically significant after accounting for conscientiousness, creativity, and visuo-motor coordination, suggesting that these traits may act as confounding factors, introducing bias in the link between artist status and reproductive success. This possibility warrants further examination.

The above-described models assume linear relationships between variables of interest. However, age had a non-linear relationship both with being an artist (younger and older men were less likely to be artists) and the number of children (younger men had fewer children, while there was little difference after the age of 40). Therefore, we additionally ran models accounting for the curvilinear relationship between age and the number of children. In these models, the relationship between being an artist and reproductive success was much weaker and not significant.

In the mediation models, we considered the possibility that conscientiousness, creativity, and visuo-motor coordination might serve as mediators in the relationship between being an artist and reproductive success. We found that artists scored higher than non-artists on conscientiousness and creativity, and that these traits were positively associated with the number of children. These results are consistent with the idea that conscientiousness and creativity could be the proximal mechanisms through which art is related to higher reproductive success. Again, however, our cross-sectional design does not allow for establishing causal directions, and further longitudinal or experimental work is needed before drawing firm conclusions about whether art is an adaptation.

In summary, the different analytical approaches – with varying controls and models – led to divergent results. Therefore, none of the analyses should be considered definitive, and conclusions depend on the chosen model and included control variables. Although it is challenging to synthesise these results, we now discuss several possible explanations and their implications.

In our study, key traits for reproductive success were creativity and conscientiousness. Recent studies confirm that creativity remains one of the top characteristics sought by people in romantic partners^67^. Thus, creativity appears to be an adaptive trait that may benefit one’s reproductive success. Interestingly, previous research has found mixed evidence regarding the link between conscientiousness and reproductive outcomes. Some studies indicated that, in general, less conscientious individuals have more offspring (e.g. in American samples:^68^), while others showed the opposite pattern of results (in Tsimane^69^ and Serbians^70^). Our data, similar to Jokela’s^71^, suggest a positive and stronger relationship between these two variables in more traditional societies.

This study has certain limitations, particularly because it was conducted over a limited period in a population that is very difficult to test (although this latter point also made it a fascinating case in relation to the hypotheses posed). One such limitation is the simplified (though commonly used) approach that we took to measure reproductive success. When considering reproductive success, it is crucial to recognise the context-dependent factors and trade-offs that shape how this concept is measured^72–74^. One important consideration is the quantity–quality trade-off, which suggests that different environments or conditions may prioritise either the number of offspring or the quality of care and survival. In our study, we only examined general data on the number of children, which serves as an indicator of reproductive success. However, future studies could refine this measure by including more specific indicators, such as the number of mates, offspring surviving to a certain age, fertility rates, offspring mortality rates, as well as their proximate determinants, such as interbirth intervals, the health of mates and offspring, and the age of spouses at marriage^75^. Incorporating these variables would provide a more nuanced understanding of the reproductive success of artists and the phenomenon under study.

Another important avenue for future research concerns the mediating role of mate access in the relationship between artistic involvement and reproductive outcomes. While our current study focused on the number of children as a general indicator of reproductive success, previous research suggests that reproductive output may be indirectly influenced by social or biological traits that increase access to mating opportunities. For instance, voice pitch in men^76,77^ or creativity^78^ have been shown to enhance access to mates, which in turn influences reproductive success. Moreover, ethnographic research in our studied population suggests that factors such as the number of spouses or differential access to fecund women may play a crucial mediating role. In future, researchers should therefore consider collecting data on the number of romantic or marital partners (past and present), marriage patterns (e.g. monogamy, polygyny, serial monogamy), and cultural norms surrounding mate choice and marriage strategies. Such data would allow researchers to explore whether artistic ability translates into reproductive benefits via enhanced access to mates, and how this dynamic may vary across societies with different mating systems and gender norms.

Another issue is that one important variable was not accounted for in the study design: the quality of the art. In other words, high-performing artists were grouped together with those whose art was of lesser quality. However, we anticipated technical challenges regarding the cost and complexity of implementing objective assessments of the participants’ artwork by external evaluators, as opposed to relying on self-assessment. We acknowledge this as a limitation of our study and hope to address it in future research.

As described in the methods section, we conducted a brief pre-test to ensure that the questions in our questionnaire were understandable to respondents. The reliability of our tool is further supported by the exceptionally high Cronbach’s alpha score for consistency. However, we did not assess the validity and reliability of our tools beyond this, and future studies of this type may benefit from additional measures to evaluate these factors. Finally, the study was conducted in Indonesian, which is understandable to our participants, although it is not their native language. Recent reports (albeit from a different field, but not to be disregarded) suggest that the language used in surveys can influence the responses obtained^79^.

To conclude the limitations section, we emphasise once again that while our study suggests certain relationships based on previously proposed models, it is not an experiment and cannot directly confirm causality. This limitation should be kept in mind when interpreting the presented results.

To conclude, the relationship between being an artist and reproductive success is more complex than initially assumed. Our findings indicate that while being an artist was initially linked to higher reproductive success, this effect diminished when controlling for key variables such as creativity, conscientiousness, and visuo-motor coordination, which may act as mediators or confounders. Additionally, the curvilinear relationship between age and reproductive success further complicates the association, suggesting that any potential effect is weak and requires cautious interpretation.

We believe our study holds significant value, as it is the first to test this relationship in a traditionally living population with natural fertility and high birth rates. The observed effects are likely so small that only a meta-analysis could definitively identify them. Even in Western societies, evidence for such a phenomenon is scarce, and the largest behavioural genetics study to date found only a very weak association (*r* = 0.09) between the number of children and musical achievements in men^34^. Additionally, the adaptiveness of art may depend on the specific context in which it is expressed, with art potentially serving adaptive functions in some environments but not in others. For example, it may be adaptive only in contexts where artistic expression contributes to increased status.

In line with prior theories, various hypotheses regarding artistic behaviour and aesthetic abilities^14,16,17,80,81^ provide complementary perspectives that are not mutually exclusive. Just as feathers evolved for thermoregulation before being adapted for flight, artistic behaviour might have begun as a by-product but later acquired evolutionary relevance through cultural or ecological factors. While our study does not seek to definitively test the adaptation and by-product hypotheses, it contributes to the understanding that the relationship between artistry and reproductive success is multifaceted, shaped by a combination of cognitive, creative, and social traits.

Further research is needed to explore these dynamics across diverse populations, as only cumulative empirical evidence can strengthen the case for adaptationist theories^82^. Ultimately, our findings emphasise the need to consider multiple interacting factors and models when studying the evolutionary significance of artistic behaviours.

## Electronic supplementary material

Below is the link to the electronic supplementary material.

Supplementary Material 1

## Data Availability

Original data created for the study are available in a persistent repository upon publication, and can be accessed: https://osf.io/tfxjd/?view_only=590af1935a124c6dbf7babba00fd77ee.
