# Supplementary material for: Testing the art as adaptation hypothesis through artistic practice and reproductive success in Papua woodcarvers
Source: Sci Rep. 2025 Jul 1;15:21102. doi: 10.1038/s41598-025-06394-y (PMC12214525; doi:10.1038/s41598-025-06394-y)
Supplement: Supplementary file 1 — Supplementary Material 1 [file 41598_2025_6394_MOESM1_ESM.docx]

Supplementary Materials

**Figure S1.**

Distribution of the number of children across the entire sample.


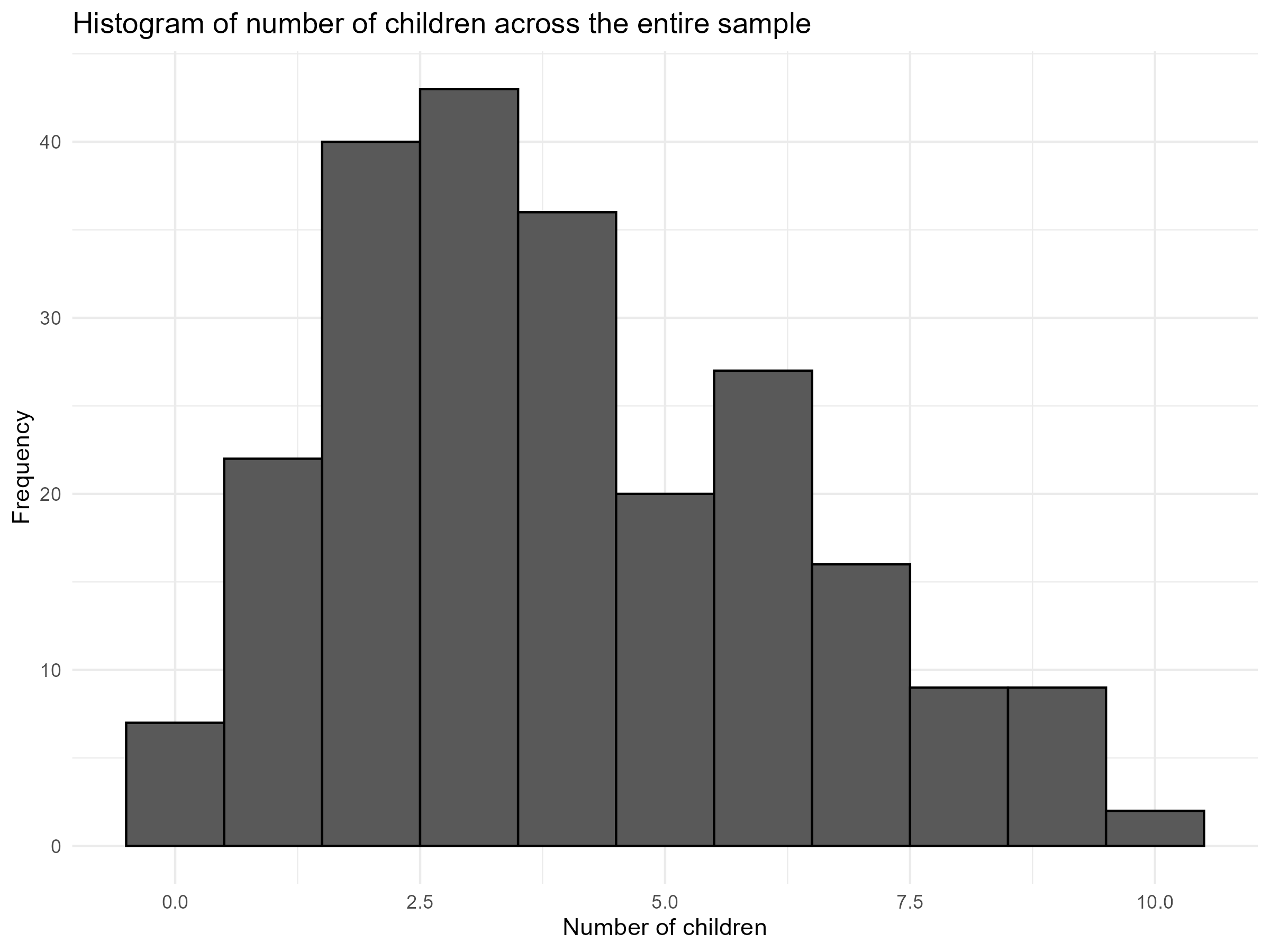


**Figure S2.**

Distribution of the number of children across artists.


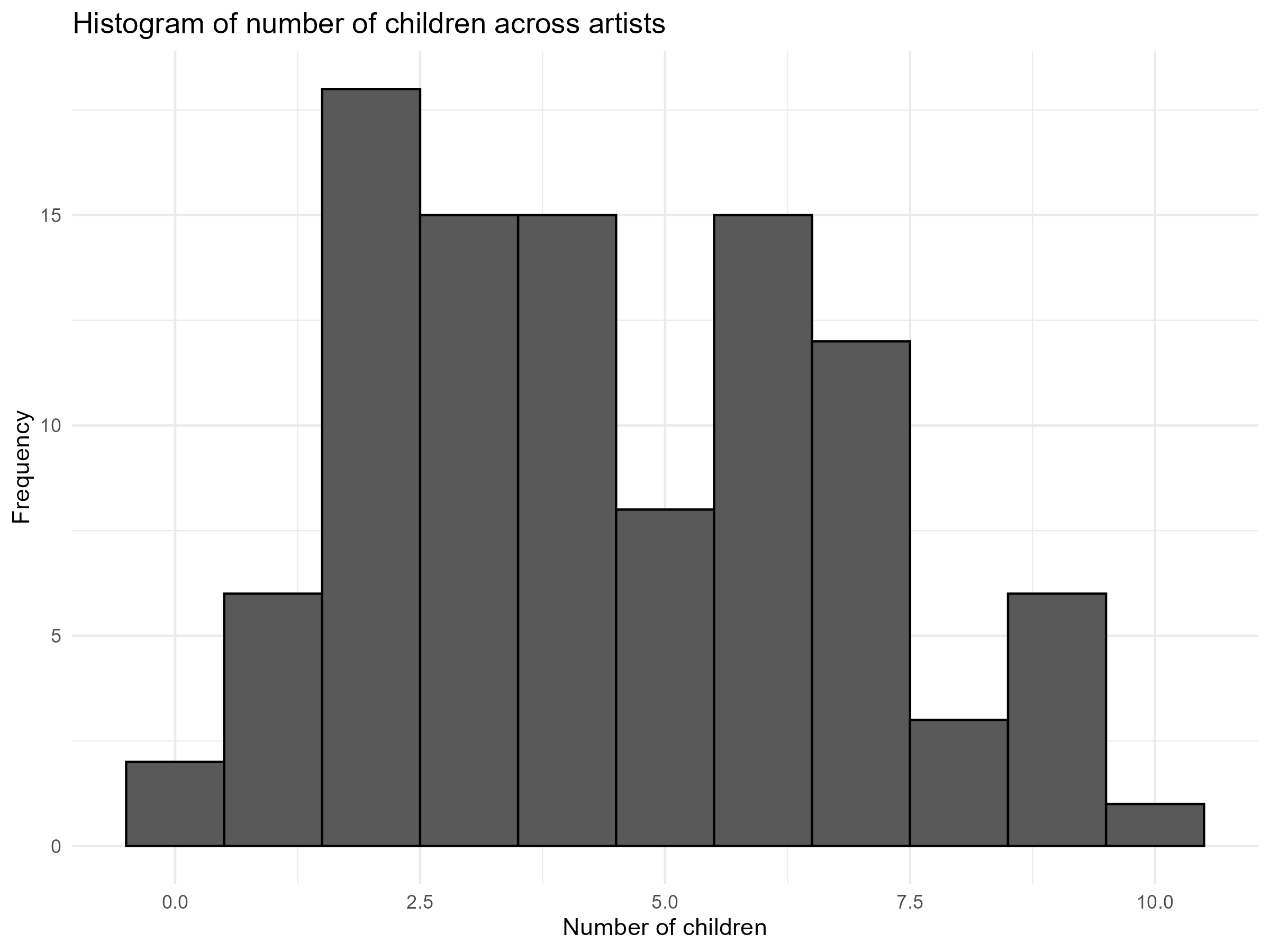


**Figure S3.**

Distribution of the number of children across non-artists.


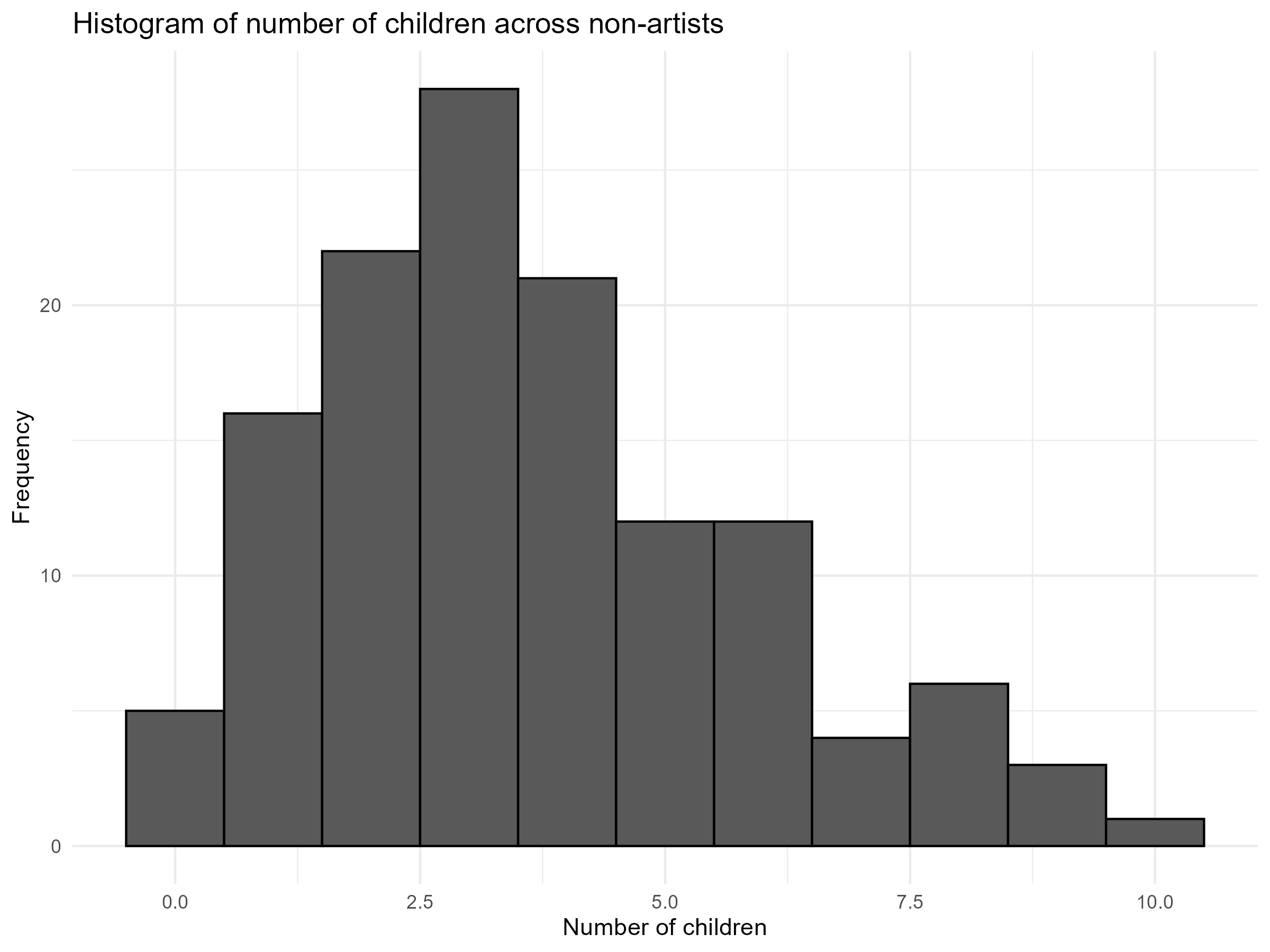


**Table S1**

The results of the linear regression models with reproductive success (i.e., the number of children) as the outcome variable and being an artist as a predictor variable in the first model, and being and artist, conscientiousness, creativity, and visuo-motor coordination as predictor variables in the second model, controlling for age and society (i.e., Asmat and Kamoro).

|  | **Models controlling for a linear relationship between the number of children and age and society** | | | | | | | | | | |
| --- | --- | --- | --- | --- | --- | --- | --- | --- | --- | --- | --- |
|  | **Adj. *r^2^* = 0.222, *F*(2,228) = 31.9, *p* < 0.001***** | | | | | **Adj. *r^2^* = 0.310, *F*(5,225) = 21.7, *p* < 0.001***** | | | | | |
| **Predictor** | ***b*** | ***β*** | ***SE*** | ***95% CI*** | ***p*** | ***b*** | ***β*** | | ***SE*** | ***95% CI*** | ***p*** |
| Being an artist ^a^ | 0.521 | 0.112 | 0.059 | [-0.004, 0.229] | 0.059 | -0.281 | -0.06 | | 0.063 | [-0.185, 0.064] | 0.341 |
| Age | 0.087 | 0.449 | 0.059 | [ 0.334, 0.565] | < .001*** | 0.095 | 0.49 | 0.055 | | [ 0.382, 0.599] | < .001*** |
| Society | 0.596 | 0.258 | 0.119 | [ 0.023, 0.493] | 0.032* | 0.715 | 0.31 | 0.112 | | [ 0.088, 0.531] | 0.006** |
| Conscientiousness ^b^ |  |  |  |  |  | 0.750 | 0.241 | 0.058 | | [ 0.127, 0.355] | < .001*** |
| Creativity ^c^ |  |  |  |  |  | 0.653 | 0.201 | | 0.061 | [ 0.081, 0.320] | 0.001*** |
| Visuo-motor Coordination ^d^ |  |  |  |  |  | 0.100 | 0.091 | | 0.057 | [-0.021, 0.203] | 0.112 |

*Note*. * *p* < 0.05, ** *p* < 0.01, *** *p* < 0.001; ^a^ – a dichotomous variable of being an artist or not (with 0 coded as non-artists and 1 as artists); ^b^ – a mean score on the conscientiousness scale); ^c^ – the total amount of generated uses for a stone; ^d^ – a mean of the right- and left-hand score on the visuo-motor coordination test.
